# Effect of the Hemin Molecular Complexes on the Structure and Properties of the Composite Electrospun Materials Based on Poly(3-hydroxybutyrate)

**DOI:** 10.3390/polym13224024

**Published:** 2021-11-21

**Authors:** Polina Tyubaeva, Ivetta Varyan, Anton Lobanov, Anatoly Olkhov, Anatoly Popov

**Affiliations:** 1 Academic Department of Innovational Materials and Technologies Chemistry, Plekhanov Russian University of Economics, 36 Stremyanny Lane, 117997 Moscow, Russia; ivetta.varyan@yandex.ru (I.V.); avlobanov@mail.ru (A.L.); aolkhov72@yandex.ru (A.O.); anatoly.popov@mail.ru (A.P.); 2 Department of Biological and Chemical Physics of Polymers, Emanuel Institute of Biochemical Physics, Russian Academy of Sciences, 4 Kosygina Street, 119334 Moscow, Russia

**Keywords:** poly(3-hydroxybutyrate), porphyrin complex, hemin, semicrystalline polymer, supramolecular structure, electrospun fibrous materials, molecular mobility

## Abstract

The creation of innovative fibrous materials based on biodegradable semicrystalline polymers and modifying additives is an urgent scientific problem. In particular, the development of biomedical materials based on molecular complexes and biopolymers with controlled properties is of great interest. The paper suggests an approach to modifying the structure and properties of the composite materials based on poly(3-hydroxybutyrate) (PHB) obtained by the electrospinning method using molecular complexes of hemin. The introduction of 1–5 wt. % of hemin has a significant effect on the supramolecular structure, morphology and properties of PHB-based fibers. Changes in the supramolecular structure intensified with the increasing hemin concentration. On the one hand, a decrease in the fraction of the crystalline phase by 8–10% was observed. At the same time, there is a decrease in the density of the amorphous phase by 15–70%. Moreover, the addition of hemin leads to an improvement in the strength characteristics of the material: the elongation at break increased by 1.5 times, and in the tensile strength, it increased by 3 times. The antimicrobial activity of the hemin-containing composite materials against *Escherichia coli* and *Staphylococcus aureus* was confirmed. The obtained materials are proposed to be used in the creation of composite systems for regenerative medicine.

## 1. Introduction

In recent years, composite polymer materials modified with metal complexes of porphyrins have been widely used in various fields: electrically conductive fibers [1,2,3], fiber-optic sensors [4,5], materials for photonics [6,7], gene therapy [8,9] or biomedicine [10,11,12]. The creation of such composites with controlled functional properties is an actual scientific direction [13,14,15]. One of the reasons for the dynamic growth of interest in metal complexes of tetrapyrrolic macrocycles is the advances in the chemical synthesis and chemistry of tetrapyrroles, which make it possible to obtain numerous analogues of natural systems (porphyrins, texaphyrins, chlorins, corroles and others) for biomedical purposes [16,17,18,19,20].

Thus, various porphyrin complexes of natural and synthetic origin can be used to modify polymer composite systems to achieve special properties and a unique combination of biomedical characteristics. Polymer-based materials are widely developed for creating such composites based on a polymer matrix and a porphyrin complex [21,22,23].

One of the most effective ways to create such materials is electrospinning [24]. This method of forming ultrathin fibers by drawing a jet of polymer solution under the action of physical forces allows for a controlled uniform introduction of additives of various nature into the polymer matrix. Success has already been achieved in different global regions in the introduction of various porphyrin complexes into the structure of nanofibers, as well as in controlling the position of the tetrapyrrole molecule [25,26,27,28,29].

Achievements in the field of creation and research of binary compositions based on biocompatible polymers have found great success. There is a high number of effective biocompatible nonwoven compositions such as poly(3-hydroxybutyrate-co-3-hydroxyvalerate)/polylactide [30], poly(butylene adipate succinate)/polylactide [31], poly(butylene adipate terephthalate)/polylactide [32] and poly(ε-caprolactone)/poly(3-hydroxybutyrate) [33].

Poly(3-hydroxybutyrate) is a promising polymer for the therapeutic applications. PHB is characterized by a high melting point, a high degree of crystallinity and low permeability to oxygen, water and carbon dioxide [34]. This biopolymer obtained from renewable sources degrades as soon as it comes under the influence of a biologically active environment [35] and is biocompatible with the human body [36]. There is a wide number of PHB-based composites for biomedicine with polyethylene glycol [37], polylactide [38], polycaprolactone [39], chitosan [40] and elastomers [41].

The combinations of PHB with small concentrations of modifying additives of various nature are of particular interest. Nanoparticles [42], carbon nanotubes [43], catalysts and enzymes [44] can be used among the components of a polymer composite. The surface modification and nanostructuring of fibrous layers are possible [45], as well as the encapsulation of bioactive molecules [46].

In this work, close attention was paid to a complex of natural origin-hemin. Due to its properties, hemin can be used in various biomedical materials as a basis for binding proteins to a polymer [47], for container molecules (such as cavitands and capsules) for delivering systems [48], for constructing new biocatalysts tailored to specific functions [49], for creation of innovative anticoagulants [50] and others. Our aim was to comprehensively study the properties of new biocompatible composites based on a system of polymer and hemin for biomedical applications. One of the most promising areas for these materials is a wound-healing bandage: biopoylmer-hemin-protein that provides regeneration. The unconditional advantages of hemin are not limited only to its natural origin and biocompatibility with a living organism [51].

Hemin has thermal stability, which is suitable for various processing conditions of the hemin-based composites [52]. Hemin is also known as a complex with some antimicrobial activity against *S. aureus* [53]. There is a large number of works that show the effectiveness of hemin as a binding peptides element used for medical purposes [54,55]. The low cost of hemin extraction and purification makes it even more attractive for research purposes and further industrial translation [56].

There are several successful works on the electrospinning of fibrous materials containing hemin [57,58] and also about various ways of combining hemin with nanofibers [59], in which the effectiveness of hemin in biomedical application is shown.

The main goal of the article was to study the influence of hemin molecular complexes on the structure and properties of composite materials based on poly(3-hydroxybutyrate). An integrated approach allows us to evaluate the mutual influence of the molecular complex of natural origin–hemin on the macromolecules of poly(3-hydroxybutyrate), as well as to use the obtained data for the directed modification of the composite structure of biomedical polymers. Various methods of evaluating the structure (microscopy, APR, DSC, SEM), mechanical and antimicrobial properties of materials were used in the work. The results obtained indicate a significant influence of the molecular complex on various properties of the polymer, especially on the supramolecular structure.

## 2. Materials and Methods

### 2.1. Materials

**Figure 1:**
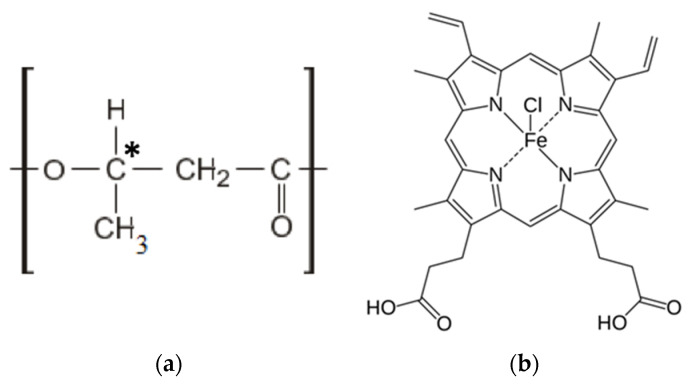
Structural formulas of PHB (**a**) and hemin (**b**).

The semicrystalline biodegradable polymer poly(3-hydroxybutyrate) (PHB) 16F series (BIOMER^®^, Frankfurt am Main, Germany) with molecular weight of 206 kDa, density of 1.248 g/cm^3^ and crystallinity level of 59% was used to prepare the samples (Figure 1a). A tetrapyrrole complex from the class of porphyrins of natural origin-hemin was used as a modifying additive. Hemin is a coordination complex of iron (oxidation state: III) (Figure 1b) [60], isolated from bovine blood (Russia).

### 2.2. Preparation of the Electrospun Materials

Polymer nanofibrous materials were obtained by electrospinning (ES) on a single-capillary laboratory unit with the capillary diameter 0.1 mm. The PHB-hemin materials were obtained by the method of double-solution electrospinning [61,62]. Finely dispersed PHB powder was dissolved in chloroform at a temperature of 60 °C in order to make the forming solutions. Hemin was dissolved in *N*,*N*-dimethylformamide at a temperature of 25 °C and homogenized with the PHB solution. The content of PHB in the solution was 7 wt. %, and the content of hemin was 1, 3 and 5 wt. % of the PHB. The voltage of the ES was 17–20 kV, the distance between the electrodes was 190–200 mm, and the gas pressure on the solution was 10–15 kg(f)/cm^2^. Electrical conductivity of the forming solution was 10–14 μS/cm, and viscosity of the hemin-PHB solution was 1.4–1.9 Pa s (viscosity of the 7% PHB in chloroform was 1 Pa s).

### 2.3. Methods

#### 2.3.1. Microscopy

Primary data of morphology and topology of the fibrous materials with different content of hemin were obtained by optical microscope Olympus BX43 (Tokyo, Japan). Scanning electron microscopy (SEM) was performed by the Carl Zeiss (Wurttemberg, Germany) in order to characterize the morphology.

#### 2.3.2. Morphology and Density Analysis

Morphology and geometry of fibers were evaluated by the counting method using software Olympus Stream Basic [25]. The average diameter of fibers was determined manually for each fiber on the z-stack at five different points of the sample excluding defective areas.

Average density characterizes the mass per unit volume of the material. The data were averaged over ten samples. The density, δ, was defined as:

(1) $$\delta =\frac{m}{l\times B\times b}$$

where m is the mass; l is the length; B is the width; and b is the thickness.

Theoretical porosity is the percentage of the mass of the material and the fiber-free volume. The data were averaged over five samples.

#### 2.3.3. The Energy-Dispersive X-ray Spectroscopy

The energy-dispersive X-ray spectroscopy (EDX) was performed by the Tescan VEGA3 (Brno, Czech Republic) in order to analyze elemental composition of the samples. The carbon, oxygen, chlorine and iron atoms were determined during the investigation. The measurements were carried out when the samples were coated with platinum.

#### 2.3.4. Differential Scanning Calorimetry

The study of the thermal properties of the samples was carried out by differential scanning calorimetery (DSC) by the Netzsch 214 Polyma (Selb, Germany), in an air atmosphere, with a heating rate of 10 °K/min and with a cooling rate of 10 °K/min. The samples were heated from 20 to 220 °C and then cooled to 20 °C twice. The average statistical error in measuring thermal effects was ±2.5%. The enthalpy of melting was calculated using the NETZSCH Proteus software. The mass of all samples encapsulated into aluminum pans was kept at about 7 mg. The thermal analysis was carried out according to the standard technique [63].

The crystallinity degree of the samples, χ, was defined from the first scan as:

(2) $$\chi =\frac{\Delta H}{H_{\mathrm{PHB}}}\times 100\%$$

where ΔH is melting enthalpy and $H_{\mathrm{PHB}}$ is the melting enthalpy of the ideal crystal of the PHB, 146 J/g [64].

#### 2.3.5. Electron Paramagnetic Resonance

Electron paramagnetic resonance (EPR) spectra (X range) were recorded on an EPR-V automatic spectrometer (Moscow, Russia). The modulation amplitude was always much smaller than the resonance linewidth and did not exceed 0.5 G. The stable nitroxyl radical TEMPO was used as the spin probe. The radical was introduced into the samples from the gas phase at 50 °C. The radical concentration in the polymer was determined using the Bruker WinEPR software (the reference was CCl4 with the radical concentration not exceeding 10^−3^ mol/L). The experimental spectra of the spin probe in the region of slow motions (τ > 10^−10^ s) were analyzed within the model of isotropic Brownian rotation using the program described in [65]. The probe rotation correlation time τ in the region of fast rotations (5 × 10^−11^ < τ < 10^−9^ s) was found based on the ESR spectra from the formula [66]:

(3) $$\tau \;=\;\Delta H+\times \;(\sqrt{\frac{I_{+}}{I_{-}}}-1)\;\times \;6.65\;\times \;10^{-10}$$

where ΔH+ is the width of the spectrum component located in a weak field and $\frac{I_{+}}{I_{-}}$ is the ratio of the component intensities in the weak and strong fields, respectively. The measurement error τ was 5%.

#### 2.3.6. Mechanical Properties

Mechanical properties (tensile strength, elongation at break) were examined by a tensile compression testing machine Devotrans DVT GP UG 5 (Istanbul, Turkey) according to Standard Test ASTM D5035-11. The data were averaged over five samples.

Elongation at break, *ε*, was defined as:

(4) $$\varepsilon =\frac{\Delta l}{l_{0}}\times 100\%$$

where Δl the difference between the final and initial length of the sample and l_0_ is the initial length of the sample.

#### 2.3.7. Antimicrobial Tests

Antimicrobial activity of the samples was studied using the strains of *Escherichia coli* O157:H7, *Candida guilliermondii* ATCC 6260 and *Staphylococcus aureus* MRSA. The lysis zone was considered as a marker of antimicrobial activity. The Petri dishes were inoculated with a standardized inoculum of test microorganisms. The samples were placed into the Petri dishes. The strain was grown in BSA/agar at 37 °C and allowed to stay with the samples for 24 h.

## 3. Results

### 3.1. Morphology of the Fibrous Material

The essence of the electrospinning process ensures the curing of the fibers of the polymer matrix after complete evaporation of the solvent [67]. In general, the structure of fibrous material produced by the ES method should be described as chaotic [68]. Long ultrathin fibers under the action of a complex of physical forces are cured and stacked, taking a certain position in the material layer. This structure should be described as highly developed in terms of a very high surface area [69]. The variation of the ES conditions, as well as the formulation of the forming solution, make it possible to influence the structural features of the produced electrospun material and even to predict the morphology of individual fibers with high accuracy. The key characteristics for a full-scale description of morphology are: density, which describes the capacity of the polymer composite material; average diameter of the fibers; theoretical porosity, expressed as a percentage the proportion of the fiber-free volume of the material, as well as pore size.

#### 3.1.1. Results of the Optical Microscopy

**Figure 2:**
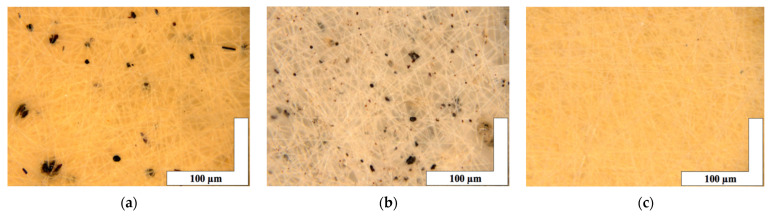
The microphotographs of PHB with different content of hemin: 1 (**a**), 3 (**b**) and 5 (**c**) wt. %.

The microphotographs of the material based on the PHB with different content of hemin are shown in Figure 2.

It is important to note that microphotographs obtained by optical microscopy methods clearly show that fibers with 1 and 3 wt. % (Figure 2a,b) of hemin have black inclusions on the surface of the fibers with average sizes 4–32 µm for 1 wt. % and 0.7–17 µm for 3 wt. %. In the case of 5 wt. % of hemin (Figure 2c), the inclusions are practically absent (1–4 µm).

It is important to stress that the introduction of hemin in the PHB solution had a significant impact on its forming properties. Even small concentrations of hemin (1–5 wt. %) made a significant contribution to the electrical conductivity of the solution increasing it due to the iron atom in the porphyrin molecules. Due to the higher electrical conductivity, the jet in the ES process was moving more organized. As a result, the fibers cured well, the pore size decreases, while increasing the porosity of the material. That is, at a lower density, the material is characterized by a large percentage of the air layer between well-cured fibers without gluing streaks and thickenings as the hemin concentration increases. The characteristics of the nonwoven materials are presented in Table 1.

#### 3.1.2. Results of the Scanning Electron Microscopy

**Figure 3:**
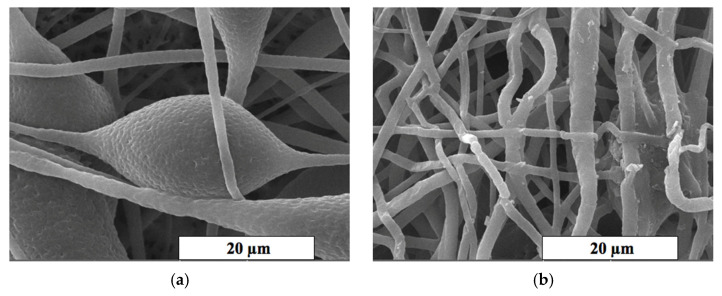

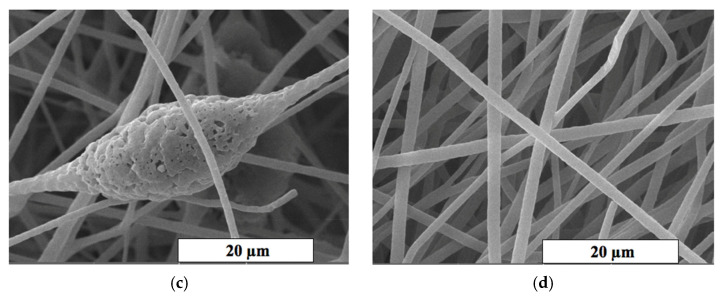
The SEM microphotographs of PHB with different content of the hemin: 0 (**a**), 1 (**b**), 3 (**c**) and 5 (**d**) wt. %.

The SEM method was used for a detailed study of the morphology of fibrous materials. The microphotographs of the most interesting areas of the material based on the PHB with different content of hemin are shown in Figure 3. It can be seen that the surface texture of the initial fibers (Figure 3a) and with a low content of hemin (less than 3 wt. %) is heterogeneous. The fibers are characterized by a greater degree of tortuosity, adhesions and the presence of large formations in the form of elliptical thickenings with a low additive content (0–3 wt. %). The average size of these structures was 20–30 µm in the longitudinal direction and 15–25 µm in the transverse direction.

An increase in the hemin concentration leads to a decrease in diameter and an increase in the uniformity of fibers. The microtopography and the structure of pores change with the introduction of even 1 wt. % of the additive (Figure 3a,b). Defects in the form of glues and thickenings are extremely rare, the fibers thin out, and the number of defects decreases with the introduction of hemin. The roughness on the surface of the fibers almost completely disappears at 5 wt. % of the additive, as well as fusiform thickenings. An area of interest is the surface of the fibers and the change in topology caused by an increase in the concentration of hemin. It is important to note that the concentration of the additive significantly affects the presence of pores, dividing them more pronounced and deep (Figure 3b,c).

### 3.2. Chemical Constitution of the Material

#### Results of the EDX

**Figure 4:**
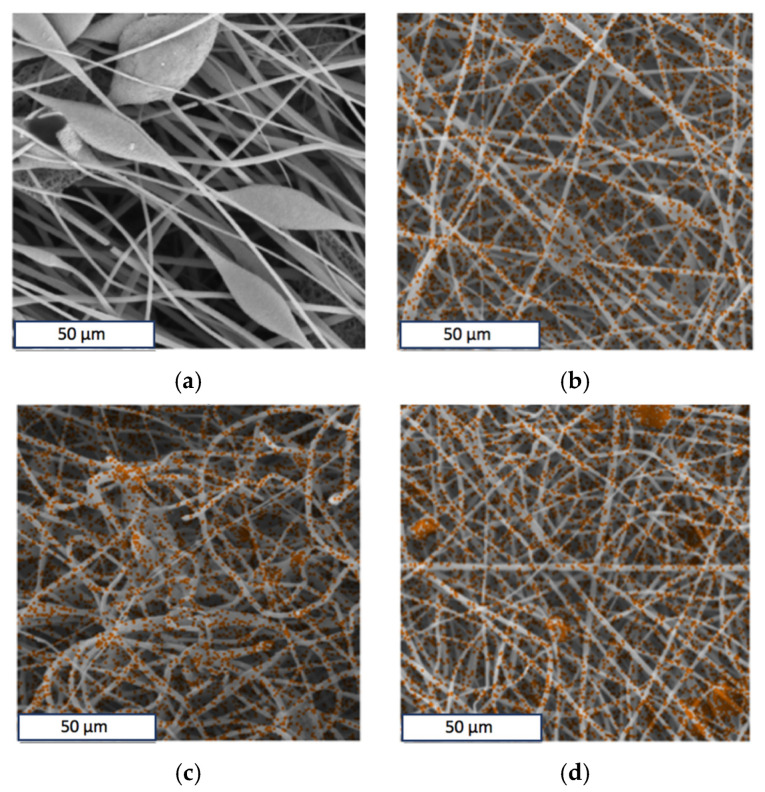
The energy-dispersive X-ray spectroscopy elemental analysis of Fe in the PHB with different content of hemin: 0 (**a**), 1 (**b**), 3 (**c**) and 5 (**d**) wt. %.

Optical micrographs show the presence of inclusions on the surface of the fibers (Figure 2a,b). The EDX method was used to determine the uniformity of hemin distribution in the PHB. The results of the analysis showed that hemin in the studied concentration region is distributed fairly evenly in the material and not only concentrated in inclusions on the surface. Iron and chlorine atoms were chosen as the atoms identifying hemin, since they are part of the central area of the tetrapyrrole ring. The results are shown in Figure 4. The results for iron and chlorine were identical; therefore, this article is given only for iron (orange color in Figure 4b–d).

### 3.3. Supramolecular Structure of the Material

The effect of hemin supplementation on the supramolecular structure of PHB was considered in the work. PHB is a semicrystalline polymer whose crystallites tend to be laid in lamellae [70]. In case of sufficient time and optimal conditions for repeated cold crystallization, the formation of regular crystal structures, including PHB spherolites, is possible [71]. The influence of production on the formation of the crystalline structure of PHB is high; therefore, it introduces boundary conditions into the process of formation of the supramolecular structure. Thus, the ES method promotes the formation of extended oriented macromolecules [72], which can be called fibrils [71].

**Figure 5:**
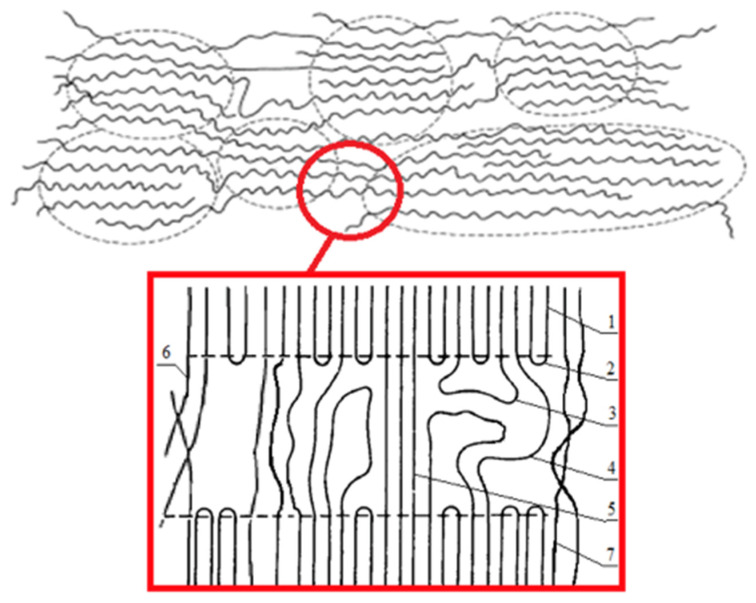
The organization of PHB macromolecules: 1—crystal regions; 2—regular chain fold; 3—irregular chain fold (free loop); 4—pass-through molecules; 5—the shortest, elongated and stressed pass-through molecules; 6—interfibrillar pass-through chains; and 7—interfibrillar structures.

Earlier, we investigated the structure of crystallites in PHB fibrils by the X-ray method [73]. The long period was 61 µm, the longitudinal size of the crystallite was 270 µm, the transverse size of the crystallites was 370 µm, the accuracy of determination was ±2 µm, the degree of crystallinity at large angles was 53% and at small angles of X-ray diffraction was 60%, and the accuracy of determination was ±5%. These results are in good agreement with the known processes occurring during the crystallization of PHB macromolecular structure [74,75]. In general, the supramolecular organization of the electrospun PHB can be represented as shown in Figure 5.

Thus, the PHB used in this work should be characterized by the following parameters: the amorphous phase in the material structure was 35–40%, and the crystalline phase was 60–65%, including organized crystallites being 35% [25].

In this paper, the effect of the hemin complex on the supramolecular structure of PHB was considered in detail.

#### 3.3.1. Crystalline Phase

**Figure 6:**
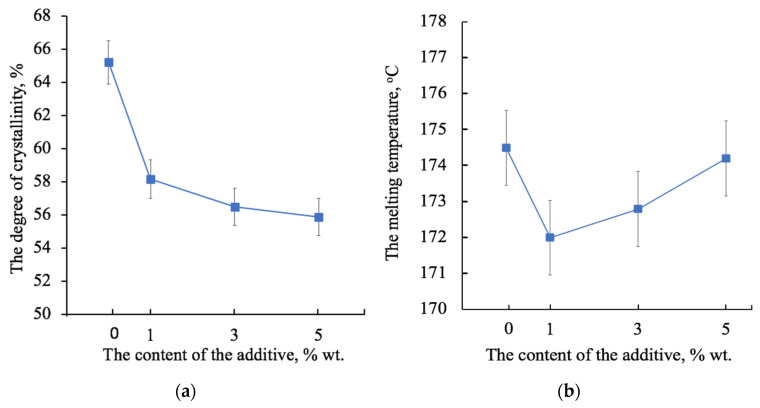
The changes in the degree of crystallinity (**a**) and the melting temperature (**b**) of the PHB depending on the concentration of the hemin from the first heating.

The degree of crystallinity is understood as the total fraction of the crystalline phase in a semicrystalline polymer, which includes both well-crystallized crystallites and uncrystallized, defective and paracrystalline formations. Figure 6 shows the changes in the degree of crystallinity (Figure 6a) and the melting temperature (Figure 6b) of the PHB after the first heating. An increase in the concentration of the additive reduces the proportion of the crystalline phase within 8% (Figure 6a), which indicates that hemin has a significant effect on the crystallization of the polymer during the solution rejection period. At the same time (Figure 6b), the melting point increases with increasing concentration. Although the difference in melting temperatures is small (2 °C), it can still be a marker of the processes occurring at the crystallization stage. Interestingly, 1 wt. % of hemin reduces the melting point. However, with an increase in the hemin content, the melting point grows. In general, this dependence is in good agreement with the dynamics of changes in the morphology of the fiber surface: with the addition of 1 wt. % (Figure 3b) and 3 wt. % (Figure 3c), the grain surface of the fibers is rough and has depressions, cavities, and pores, which are definitely smaller with 5 wt. % of the additive.

**Figure 7:**
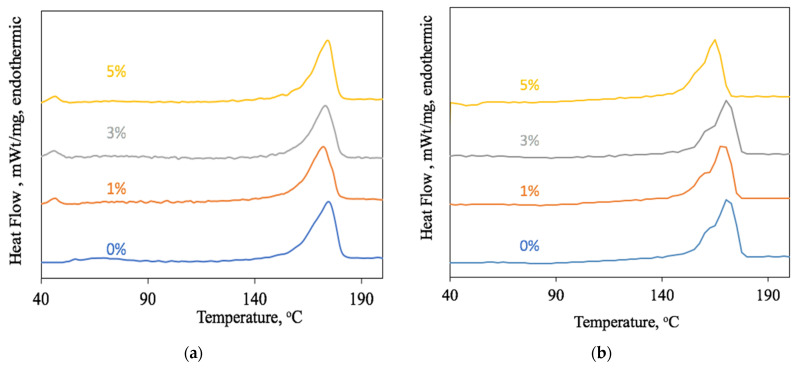
The PHB melting peaks with different hemin content: first heating (**a**) and second heating (**b**).

Of great interest is the change in the shape of the melting peak of the PHB with a different amount of hemin. During the first heating (Figure 7a), it is shown that the peak becomes more symmetrical with an increase in the hemin concentration, and the crystallinity decreases evenly within 10%. In the first stage of the melting process, the less organized crystallites and paracrystalline structures melt faster. It is known that these structures are formed if the PHB does not have sufficient capacity for slow crystallization [76]. The introduction of the hemin into the solution affects the course of crystallization. Perhaps, hemin acts as a nucleating agent of crystallization. PHB structures grow more uniform and organized, although the total number of crystal structures decreases. This effect is especially clearly seen during the second heating (Figure 7b). The low temperature shoulder from 150 to 160 °C was shown to indicate the melting of a large number of irregular crystalline formations. With an increase in the concentration of hemin, their number decreases. That is, the rate of crystallization no longer contributes to the organization of the crystalline phase of PHB.

#### 3.3.2. Amorphous Phase

**Figure 8:**
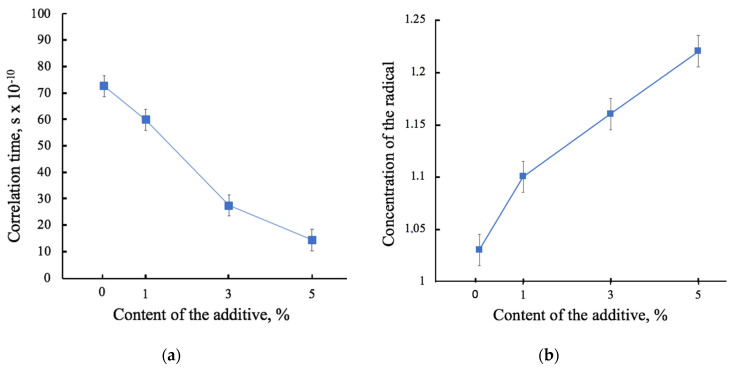
The dependence of the characteristics of the amorphous phase of PHB on the hemin concentration: spin probe correlation times (**a**) and the concentration of the spin probe in relation to the mass of the material sample (**b**).

The EPR method allows one to assess the state of the amorphous phase. The results of the EPR are in good agreement with the results of the DSC. The correlation time of the probe indicates the structure of the amorphous phase. Figure 8a shows the results of the analysis of the mobility of the spin probe TEMPO as a function of the hemin concentration. The concentration of the spin probe in relation to the mass of the material sample (Figure 8b) shows that hemin does not prevent the penetration of a radical into the material, confirming the theory of a change in the structure of the amorphous phase. That is, the more the radical enters, the less its mobility becomes. This is certainly due to the localization of the tetrapyrrole ring in the amorphous phase.

All these changes significantly affected the physical and mechanical characteristics, morphology of the composites and their operational properties. The localization of the porphyrin complexes is concentrated in the amorphous phase of the material.

### 3.4. Physical and Mechanical Properties

The physical and mechanical properties of composite materials are an important class of operational properties, but they are also to a large extent an indicator of the state of the polymer-additive molecular system. Due to changes in the supramolecular structure and morphology, there is a change in physical and mechanical properties. The results of the physical and mechanical tests are shown in Table 2.

It is important to note that at 5% hemin in the material, the tensile strength increases to 5.5 MPa, which is 3 times higher than the strength of brittle nonwovens based on PHB without additives. The elongation at break increases to 6%, which is 1.5 times higher than the one of the original PHB. However, at lower concentrations of hemin, such a high increase cannot be achieved.

### 3.5. The Antimicrobial Tests

As discussed earlier, the antimicrobial activity of hemin is known from the literature, although the short-term effect is noted [53]. The samples for the experiment were kept for 90 days from the date of production. Earlier, it was found that the fibrous materials based on PHB containing complexes of synthetic porphyrins may be unstable: there is a loss of mass, a decrease in the degree of crystallinity, and a change in physical properties that indicated the degradation of the material [25]. This process was most active within 30–60 days from the moment of production. Thus, it was planned to establish antimicrobial properties for a long period of time sufficient for the application of the composite in biomedicine.

**Figure 9:**
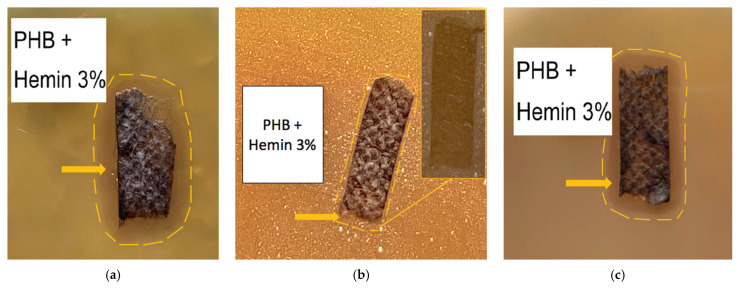

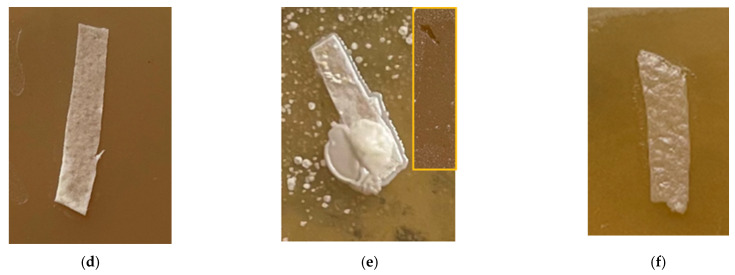
The microbiological tests of the PHB + 3% of the hemin samples against (**a**) *E. coli*; (**b**) *C. guilliermondii* (**c**) *S. aureus*; the lysis zone is shown in colored line and PHB + 0% against (**d**) *E. coli*; (**e**) *C. guilliermondii*; and (**f**) *S. aureus* as the control.

The results of the antimicrobial test are shown in Figure 9.

Figure 9 shows the results of the antimicrobial tests for the PHB materials with 3% of hemin. As follows from Figure 9, the samples of the material develop an inhibition zone around and beneath the samples. Figure 9a shows that the lysis zone increases in the zone with a larger sample thickness and therefore with a higher hemin content. In Figure 9b, (sharpness increased in comparison with the original) an insignificant lysis zone is visible, although when the sample is removed (original color, yellow frame), it is clear that the reproduction of microorganisms is stopped under the sample. It was probably discovered due to anaerobic microorganisms, although this effect was much less pronounced under PHB (Figure 9e). Figure 9c (original color) shows the lysis zone around and under the sample.

In general, the data obtained suggest that in the case of creating an antimicrobial wound-healing dressing, the inclusion of hemin in the composition positively affects the ability of the material to suppress the viability of bacteria and fungi.

## 4. Discussion

PHB is a semicrystalline polymer. The production method makes a significant contribution at all levels of polymer and fiber organization [77,78]. ES has a gradual effect both on the supramolecular structure, on the morphology of individual fibers and on the entire material as a whole. Therefore, it is customary to allocate three levels of structural organization for electrospun polymeric materials: Macroscopic (whole system), mesoscopic (fiber contact area), microscopic (structure of the fiber) scale [79]. The obtained data show that the introduction of hemin molecules has a significant impact on the organization of PHB at all levels of the organization.

Even at the stage of the ES process, the introduction of a modifying additive at low concentrations (1, 3, and 5 wt. %) can have a significant effect on the formation of the material’s structure.

It is known that ES allows for a uniform arrangement of additives that occupy the free space between the crystallites in the amorphous regions of the polymer [77]. That was confirmed by the EDX method. Hemin contains an atom of a metal in the center of the tetrapyrrole ring which certainly increased the electrical conductivity of the forming solution [78], without reducing its viscosity, which leads to an improvement in the forming properties of the ES. With the increasing in the hemin concentration, the fibers become more uniform. The ES process is stable, without the formation of smudges, drops and thickenings, which has a positive effect on the morphology of the fiber. It is also important that the fibers have time to completely cure at stage of the Taylor cone formation without forming glues in the mesoscopic material layer [66]. It gives an additional degree of variance in the application of mechanical stress to the fibers in the material. 

Figure 2 shows optical microphotographs where the black inclusions on the surface of the fibers are noticeable. The analysis using the EDX method showed that these inclusions are the hemin particles. It is important to note that with the increase in the hemin concentration, the number and size of black inclusions decrease. The nature of these inclusions could be explained by the insolubility of hemin in chloroform, the main solvent of the system. A lower concentration of hemin leads to a greater proportion of chloroform and PHB in the forming solution. This leads to the formation of agglomerates during the curing of the solution.

As a result of a more organized jet movement in the ES process, the pore area first increases by more than 6 times at 1 wt. %, and then, the difference between pure PHB and PHB with 5 wt. % of hemin reaches an excess of 3.5 times. At the same time, the porosity of the material increases from 80% to 94%. That is, at a lower density, the material is characterized by a large percentage of air space between well-cured fibers without gluing, smudges and thickening as the hemin concentration increases.

At the same time, high attention should be paid to the results of SEM. The deepening of the pores on one of the thickening of the fiber (Figure 3b,c) illustrates the effect of hemin on the morphology and topology of the fiber surface. This process is coordinated with the loosening of the supramolecular structure. This phenomenon can be explained by a more intense evaporation of the solvent in view of the electroforming technology using two solvents, which is used, among other things, to produce highly porous fibers [67]. However, this factor does not contribute to a 5 wt. % in a view of a more optimal balance of electrical conductivity and viscosity of the forming solution. In all likelihood, the presence of large hemin molecules in the intermolecular space of the PHB leads to an increase in the free volume of amorphous regions, which increases the rate of solvent desorption. Thus, achieving a balance in the molding solution allows the most homogeneous surface of thin and uniform fibers.

Of course, these changes are closely related to the supramolecular structure of PHB [77]. ES is accompanied by a high rate of deformation of the polymer solution (about 103 s^−1^) [77], that leads to a stretching and changing orientation of the polymer chains in the solution [69]. In light of the action of the ES conditions, the PHB macromolecules assume a preferential orientation, which can be conditionally called a fibrilla [71]. In our case, the PHB fibrilla is an alternation of sections of the crystalline and amorphous phases oriented along the axis with separate regions, which are described in detail in Figure 5. Such a preferential orientation causes a number of physical and mechanical properties of PHB fibers. The phenomenon of secondary crystallization of the produced PHB is known in the literature [80]. Without the influence of external forces, the PHB tends to form spherulites; however, if it does not have such a possibility, for example, in the case of pressing, pulling, forming from solution and melt, then the supramolecular crystalline phase has an incomplete character and is characterized by a tendency to a secondary crystallization [81]. In addition, with a lack of time for a secondary crystallization, more defective and paracrystalline formations are detected, as well as a fine-crystalline fraction of PHB [82]. However, introducing of the additive leads to an introduction of the crystallization centers into the polymer system, which allow the macromolecule to take a more advantageous position, which allows the crystalline phase to form better organized structures. This phenomenon was detected in the changes of the supramolecular structure of the PHB-hemin system. It can be seen from the DSC (Figure 6b) that changes in the melting temperature indicate that an increase in the hemin concentration leads to the formation of larger, regular crystallites that melt at a higher temperature, as in the absence of an additive. Probably, hemin can act as the center of crystallization of the PHB. Apparently, the clusters of hemin recorded by the EDX method (Figure 4) could be the nuclei of crystallization, initiating and accelerating the uniform growth of crystallites in the volume of the polymer phase, although the number of these crystallites decreases. Crystallinity decreases with the introduction of hemin (Figure 6a), but at the same time, the quality of the crystalline phase increases.

When discussing the DSC results in more detail, the nature of the melting processes should be noted. During the first melting (Figure 7a), the fibrillar structure melts. At the second melting (Figure 7b), the structure, which has already been decomposed and quickly crystallized during the first cooling, melts. During the second melting in the temperature range of 150–160 °C, the low-temperature shoulder of the melting peak can be seen. It characterizes the PHB mesophase or paracrystalline formations, which become smaller with increasing the hemin content, which confirms the assumption of the role of hemin as a center of crystallization.

These results are in a good agreement with the results of the EPR method (Figure 8). With an increase in hemin content, the correlation time decreases (Figure 8a), which means that the mobility of the spin probe increases. Moreover, an increasing concentration of the radical enters the sample of the material, which means that this characteristic is reliable (Figure 8b).

The described changes in the supramolecular structure also affect the mechanical properties of materials. There are two components that cause the growth of physical and mechanical characteristics: Firstly, the contribution of hemin to the organization of the supramolecular structure. Better organized macromolecules certainly lead to an improvement in the properties of the material. Secondly, the contribution of hemin affects the forming properties. The increase in the concentration of the additive leads to the formation of well-cured fibers. These fibers form the material without gluing and defects, increasing the mutual mobility of the fibers in the layer of nonwoven material, which contributes to the growth of mechanical stress, which it withstands until irreversible destruction.

The antimicrobial tests show antimicrobial activity against drug-resistant and Gram-negative *E. coli* and Gram-positive *S. aureus*. The lysis zone around the viable *C. guilliermondii* was small, although the material impeded the life of the culture with a hollow layer, which is an absolute advantage for practical use in wound-healing dressing.

## 5. Conclusions

The effect of the hemin molecular complexes on the structure and properties of the composite materials based on PHB was investigated. The possibility of optimizing methods for obtaining fibers with higher characteristics, including mechanical properties and antibacterial activity, was shown. The introduction of 1–5 wt. % of hemin has an effect on the supramolecular structure, morphology and properties of PHB-based fibers due to crystallization processes occurring at the stage of forming and curing of the fiber. The metal atom (trivalent iron) contained in the tetrapyrrole ring of the hemin makes it possible to obtain an optimal balance of electrical conductivity and viscosity for forming defect-free uniform hardened fibers at concentrations of hemin above 3 wt. %. When the hemin concentration reaches 5 wt. %, defects and pores disappear almost completely, as there is a necessary and sufficient balance of forming properties of the solution. At the same time, the degree of crystallinity of PHB decreases by 10%, consistent with a decrease in the density of the amorphous phase by 3.5 times, and the formation of more organized crystal structures occurs even with fast cooling. The addition of 5% of hemin leads to an increase in the elongation at break by 1.5 times and in the tensile strength by 3 times. Moreover, the surface morphology of the fiber changes significantly, and defects on the surface and in the interfiber space disappear. This observation serves as a basis for the modification and directional design of the supramolecular structure of a semicrystalline polymers and properties of the fibrous material. The antimicrobial activity of the composition PHB-hemin is confirmed by the characteristic of the lysis zone found in the work.

In light of the fact that hemin is widely used in medicine, as an independent drug complex in the treatment of porphyria, requiring a carrier polymer, and in various fields of creating combined drugs based on proteins and peptides for drug delivery, the combinations of PHB-hemin should definitely be recommended for use in biomedicine in view of the stability and high physical and mechanical properties of this composite.

## Figures and Tables

**Table 1:** Average values of the characteristics of the nonwoven materials based on PHB with different content of hemin.

Sample	Density, g/cm^3^ (±S.D., *n* = 10)	Average Diameter, µm (±S.D., *n* = 100)	Pore Size, µm (±S.D., *n* = 50)	Porosity, % (±S.D., *n* = 50)
PHB 0 wt. %	0.30 ± 0.01	3.50 ± 0.08	15 ± 10	80 ± 2.0
PHB with 1 wt. % of hemin	0.20 ± 0.02	2.06 ± 0.07	109 ± 10	92 ± 1.5
PHB with 3 wt. % of hemin	0.20 ± 0.01	1.77 ± 0.04	83 ± 10	92 ± 1.5
PHB mats with 5 wt. % of hemin	0.17 ± 0.01	1.77 ± 0.04	52 ± 10	94 ± 1.2

**Table 2:** Results of the physical and mechanical tests of the samples with different content of the hemin.

Sample	Tensile Strength, Mpa Δ ± 0.02 MPa	Elongation at Break, % Δ ± 0.2%
PHB with 0 wt. %	1.7	3.6
PHB with 1 wt. % of hemin	0.7	4.7
PHB with 3 wt. % of hemin	1.9	4.7
PHB with 5 wt. % of hemin	5.5	6.1

## Data Availability

Data sharing not applicable.
